# FFformer: A Lightweight Feature Filter Transformer for Multi-Degraded Image Enhancement with a Novel Dataset †

**DOI:** 10.3390/s25216684

**Published:** 2025-11-01

**Authors:** Yongheng Zhang

**Affiliations:** State Key Laboratory of Networking and Switching Technology, Beijing University of Posts and Telecommunications, No. 10 Xitucheng Road, Haidian District, Beijing 100876, China; zhangyongheng@bupt.edu.cn

**Keywords:** complex-scene image enhancement, multi-type degradation dataset, Feature Filter Transformer, Gaussian-filter self-attention

## Abstract

Image enhancement in complex scenes is challenging due to the frequent coexistence of multiple degradations caused by adverse weather, imaging hardware, and transmission environments. Existing datasets remain limited to single or weather-specific degradation types, failing to capture real-world complexity. To address this gap, we introduce the Robust Multi-Type Degradation (RMTD) dataset, which synthesizes a wide range of degradations from meteorological, capture, and transmission sources to support model training and evaluation under realistic conditions. Furthermore, the superposition of multiple degradations often results in feature maps dominated by noise, obscuring underlying clean content. To tackle this, we propose the Feature Filter Transformer (FFformer), which includes: (1) a Gaussian-Filtered Self-Attention (GFSA) module that suppresses degradation-related activations by integrating Gaussian filtering into self-attention; and (2) a Feature-Shrinkage Feed-forward Network (FSFN) that applies soft-thresholding to aggressively reduce noise. Additionally, a Feature Enhancement Block (FEB) embedded in skip connections further reinforces clean background features to ensure high-fidelity restoration. Extensive experiments on RMTD and public benchmarks confirm that the proposed dataset and FFformer together bring substantial improvements to the task of complex-scene image enhancement.

## 1. Introduction

In outdoor applications such as autonomous driving, security surveillance, and disaster response, high-quality visual imagery is essential for reliable decision-making and system performance. However, images captured in these scenarios are frequently corrupted by multiple coexisting factors, including adverse weather, environmental conditions, and hardware limitations. This reality underscores the need for robust image enhancement methods capable of handling diverse degradations to improve visual quality under real-world conditions.

Existing image enhancement datasets can be broadly divided into two categories. The first category targets a single degradation type, e.g., haze [1,2], rain [3,4], blur [5,6], or noise [7]. While these datasets enable effective restoration of the targeted distortion, they often fail when confronted with multiple co-occurring degradations, limiting their applicability in complex scenarios. The second category focuses on compound weather degradations; examples include BID2a and BID2b [8], which encompass rain, snow, and haze. While datasets like BID2 [8] represent a significant advance for restoring compound weather degradations (e.g., rain, haze, snow), their scope is primarily confined to these meteorological phenomena. They omit other critical degradation types prevalent in outdoor imaging, such as blur (from motion or defocus) and noise (from low-light or sensor limitations). Furthermore, the real-world images in these benchmarks typically exhibit only a single dominant degradation type, which does not fully capture the complex, multi-faceted degradation encountered in practice. These gaps underscore the urgent need for a dataset that covers the full spectrum of degradations encountered in outdoor scenes.

Early image enhancement methods have demonstrated effectiveness on individual tasks, e.g., dehazing [1,2], deraining [3,4], and deblurring [5,6], yet they generally fail when confronted with multiple, superimposed degradations typical of extreme outdoor conditions. More recent universal frameworks achieve promising results on diverse single degradations, but they still inadequately handle the complex mixtures of distortions found in challenging real scenes. Consequently, specialized solutions tailored to such harsh environments are essential for reliable visual information recovery.

**Figure 1:**
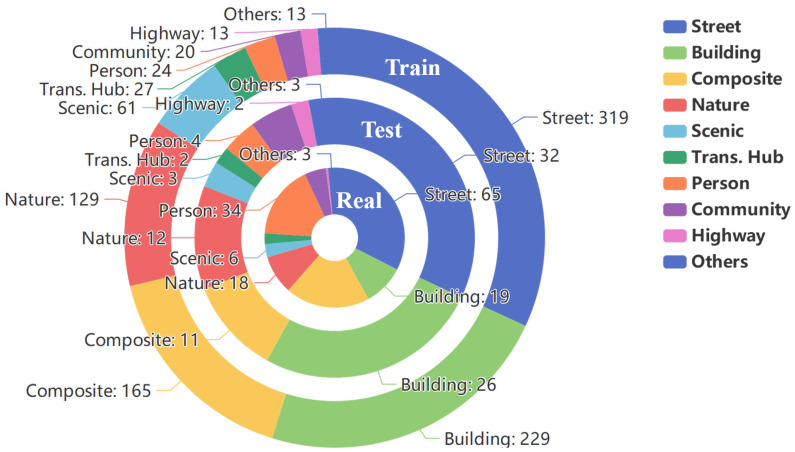
Nested doughnut charts illustrating the category distribution across the Train, Test, and Real-world subsets. From outer to inner, the rings represent the Train, Test, and Real subsets, respectively. The chart visualizes the proportion of the ten outdoor scene categories within each subset. The numerical values annotated on the charts indicate the exact number of images for each category.

To address these issues, this paper introduces the Robust Multi-Type Degradation dataset (RMTD)—a large-scale benchmark designed for outdoor image enhancement under multiple degradations—and proposes a dedicated solution, the FFformer. RMTD narrows the gap left by prior datasets by integrating a wide range of scene categories and degradation types, thereby enabling a comprehensive evaluation of enhancement robustness. Specifically, RMTD contains 10 outdoor scene classes—street, building, composite scene, natural landscape, scenic spot, transportation hub, person, residential area, highway, and others (Figure 1)—spanning urban to natural environments to ensure broad applicability. The dataset incorporates eight degradation types that reflect both meteorological and imaging-system challenges: light haze, dense haze, light rain, heavy rain, Gaussian blur, motion blur, Gaussian noise, and shot noise. These degradations are prevalent in outdoor imagery and particularly detrimental to visual quality, ensuring RMTD captures the most critical distortions observed in complex scenes. In total, RMTD provides 48,000 synthetic image pairs, each consisting of a multi-degradation image and its corresponding high-quality ground truth, making it the largest multi-degradation enhancement dataset to date. Additionally, 200 real-world multi-degradation images collected from the Internet form an extra test set for evaluating model robustness under authentic outdoor conditions.

To facilitate downstream evaluation, RMTD further furnishes over 3000 object-level annotations across 10 categories (car, truck, bus, person, motorcycle, backpack, handbag, bicycle, traffic light, and umbrella). These annotations enable assessment of enhancement algorithms from the perspective of object detection—a critical consideration for applications such as autonomous driving and surveillance. By integrating image-enhancement evaluation with detection performance, RMTD offers a comprehensive benchmark for validating the practical utility of enhancement techniques in real-world deployments.

The FFformer is an efficient and lightweight image enhancement model designed for complex scenes. It incorporates a Gaussian-Filtered Self-Attention (GFSA) mechanism and a Feature-Shrinkage Feed-forward Network (FSFN), which collectively address low-quality images with compounded degradations. Both GFSA and FSFN can not only effectively remove redundant noise introduced by different degradations but also reduce the computational cost of the model.

In the image encoder of FFformer, a Scale Conversion Module (SCM) is introduced, which enhances the features from different encoder layers and normalizes the scale of each layer. The features of these layers are aggregated through a Feature Aggregation Module (FAM) and then fed into the background decoder. Moreover, the Feature Enhancement Block (FEB) in the residual structure further strengthens FFformer’s ability to extract and enhance clear background features that are unaffected by degradations.

The key contributions of this paper can be summarized as follows:

- We propose the RMTD dataset, the first large-scale comprehensive multi-degradation benchmark including both synthetic and real degraded images, providing valuable resources for complex-scene image enhancement research.
- We introduce the FFformer, an efficient image enhancement model based on Vision Transformers (ViT), which effectively removes redundant features from compounded degradations through its GFSA mechanism and FSFN.
- An SCM and an FAM are introduced in the image encoder to fully utilize the features of different-scale layers. Meanwhile, an FEB is integrated into the residual structure of the decoder to reinforce the clear background features.
- Extensive experiments on RMTD and other synthetic and real image datasets demonstrate that FFformer achieves leading performance in various complex scenes, proving the effectiveness of the proposed dataset and method in complex scene image enhancement.

## 2. Related Work

### 2.1. Image Restoration Datasets

Most existing image restoration datasets predominantly focus on specific types of degradation, such as rain [3,4], haze [1,2], snow [9], and blur [5,6]. Some desnowing datasets [10,11] include haze as a supplementary factor. However, real extreme weather conditions often involve multiple degradation factors occurring simultaneously, such as rain, haze, and blur.

Han et al. [8] proposed the Blind Image Decomposition task, which consists of two sub-tasks: Real Scenario Deraining in Driving and Real Scenario Deraining in General. This innovative task requires the simultaneous removal of rain, haze, and snow from a single image. To address this challenge, they collected clear background images and degradation masks for rain, haze, and snow from existing restoration datasets. The resulting datasets, named BID2a (driving scenario) and BID2b (general scenario), are significant for tackling multiple degraded image restoration tasks. However, the BID2 dataset has several limitations that our work aims to address. First, its degradation coverage is limited to weather effects (rain, haze, snow) and does not include other common types like blur or noise (see Table 1). Second, the real-world test set (BID2b) largely contains images with a single dominant weather degradation, failing to reflect the challenging scenario where multiple degradations co-exist. Additionally, BID2 lacks annotations for high-level computer vision tasks, which limits its utility for evaluating the impact of restoration on downstream applications. Moreover, the baseline Blind Image Decomposition Network (BIDeN), designed specifically for these datasets, features a simplistic structure that limits its versatility and generalizability.

To overcome these limitations, we introduce the RMTD dataset, which comprises both synthetic and real images affected by multiple degradations. Unlike existing datasets, RMTD captures the diverse degradation factors encountered in real multi-degraded scenarios. It serves as a comprehensive benchmark for evaluating image restoration methodologies within the challenging context of multi-degraded conditions.

### 2.2. Image Restoration Methods

#### 2.2.1. Specific Degraded Image Restoration

Early strategies for image restoration primarily focused on addressing individual degradations through corresponding a priori hypotheses [12,13,14,15]. For example, He et al. [12] introduced the dark channel prior (DCP) to estimate transmission maps and global atmospheric light for dehazing images, based on the observation that at least one channel in a patch has values close to zero. To mitigate potential loss of detailed information in the guidance image, Xu et al. [16] developed a refined guidance image for snow removal. Li et al. [14] utilized layer priors to effectively eliminate rain streaks, offering a robust solution for rain removal. Pan et al. [17] integrated the dark channel prior into image deblurring, while subsequent studies [18,19,20] further refined and enhanced the efficiency and performance of the DCP method.

The emergence of convolutional neural networks (CNNs) and visual transformers has ushered in a new wave of learning-based image restoration methods, yielding impressive results [4,9,11,21,22,23,24,25,26]. For instance, Li et al. [24] employed a depth refinement network to enhance edges and structural details in depth maps, leveraging a spatial feature transform layer to extract depth features for dynamic scene deblurring. Jiang et al. [27] tackled the image deraining problem by developing a Multi-Scale Progressive Fusion Network, demonstrating efficient and effective deraining capabilities. Additionally, Chen et al. [11] proposed the Hierarchical Decomposition paradigm within HDCWNet, offering an improved understanding of various snow particle sizes.

#### 2.2.2. General Degraded Image Restoration

Diverging from approaches focused on specific degraded images, several methods exhibit versatility in addressing multiple degradations, including challenges such as haze, rain, and noise [28,29,30,31,32,33]. For instance, Zamir et al. [28] introduced MPRNet, which employs a multi-stage architecture to progressively learn restoration functions for degraded inputs. Wang et al. [30] proposed U-Shaped Transformer (Uformer), leveraging a locally enhanced window (LeWin) Transformer block that performs non-overlapping window-based self-attention, thereby reducing computational complexity on high-resolution feature maps. Patil et al. [31] presented a domain translation-based unified method capable of simultaneously learning multiple weather degradations, enhancing resilience against real-world conditions. Additionally, Zhou et al. introduced an Adaptive Sparse Transformer (AST) designed to mitigate noisy interactions from irrelevant areas through an Adaptive Sparse Self-Attention block and a Feature Refinement Feed-forward Network. For handling unknown or mixed degradations, DAIR [34] proposes an implicit degradation prior learning framework. It adaptively routes and restores features based on degradation-aware representations inferred directly from the input, enhancing robustness in complex real-world scenarios.

While these methods effectively manage various degradation types within a unified framework, they often struggle when multiple degradation factors coexist simultaneously. To address this challenge, Han et al. [8] proposed the novel task of Blind Image Decomposition and introduced the BIDeN as a robust baseline. Building on this foundation, we propose the FFformer for multi-degraded image restoration, demonstrating effectiveness in removing degradations such as rain, haze, noise, and blur across diverse scenarios.

### 2.3. ViT in Image Restoration

The introduction of ViT into the field of image restoration has garnered considerable attention, owing to their ability to comprehend extensive dependencies within images. This capability is crucial for discerning the broader context and relationships among various image components. Recent research has highlighted the efficacy of ViT across a diverse range of image restoration applications [30,35,36,37,38,39].

Innovatively, Liang et al. [36] proposed SwinIR, a method for image restoration based on the Swin Transformer. SwinIR leverages multiple residual Swin Transformer blocks to effectively extract deep features for various restoration tasks. Similarly, Zamir et al. [35] introduced Restormer, a hierarchical encoder–decoder network constructed with Vision Transformer blocks. Restormer incorporates several key design elements in its multi-head attention and feed-forward network to capture long-range pixel interactions, making it applicable to large images. As a result, Restormer has demonstrated exceptional performance in restoring images affected by various degradation types. To enhance ViT efficiency for restoration, MG-SSAF [40] introduces a Spatially Separable MSA module that approximates global attention with linear complexity, coupled with a Multi-scale Global MSA module to maintain cross-window interactions, offering a lightweight yet effective design.

The integration of Vision Transformers into image restoration methodologies signifies a substantial advancement in utilizing transformer-based architectures to enhance the visual fidelity of degraded images. These developments underscore the adaptability and effectiveness of ViT in capturing intricate dependencies, ultimately facilitating superior image restoration outcomes.

### 2.4. Feature Enhancement in Multi-Modal and Multi-Scale Learning

The paradigm of feature enhancement extends beyond the domain of image restoration, serving as a fundamental technique to improve data quality and model representation power across various visual tasks. Recent research demonstrates its critical role in fusing information from different modalities or scales.

For instance, in the context of intelligent transportation systems, a trajectory quality enhancement method [41] leverages onboard images to calibrate and refine vehicle trajectory data. This work exemplifies how visual features can act as a high-fidelity prior to enhance the precision and reliability of another data modality (trajectory), showcasing a cross-modal feature enhancement strategy.

Similarly, in hyperspectral image (HSI) classification, an enhanced multiscale feature fusion network [42] highlights the importance of integrating features at different scales. By designing dedicated modules to aggregate contextual information from multiple receptive fields, it significantly boosts classification accuracy, underscoring the pivotal role of multi-scale feature fusion.

While the applications differ, these works share a common thread with our proposed FEB and FSFN modules: the core principle of actively guiding the model to reinforce more informative features. Our approach aligns with this philosophy but is specifically tailored for the challenge of image restoration under multiple degradations. The FEB enhances features across the encoder–decoder hierarchy to bridge the semantic gap, while the FSFN employs soft-thresholding to sparsify and purify features. Together, they form a cohesive feature enhancement framework within our FFFormer, dedicated to suppressing degradation artifacts and recovering clean image content.

## 3. The Robust Multi-Type Degradation Dataset

The RMTD dataset consists of 48,000 synthetic multi-degraded image pairs and 200 real-world images, designed to benchmark robust image restoration models across various degradation conditions. The synthetic dataset is divided into three subsets: Train (16,000 images), Test (1600 images), and Others (30,400 images). The Others subset is used as a flexible resource, which can be employed for validation or to supplement the training set, depending on the model’s specific needs. The Real subset, containing 200 real-world images, offers an additional test set for evaluating model performance in uncontrolled, real-world scenarios. Table 1 compares RMTD with existing datasets.

### 3.1. Dataset Construction

This section describes the methodology used to construct the dataset, including the synthesis of multi-degraded images, the collection of real-world degraded images, and the object detection annotation process.

#### 3.1.1. Synthetic Multi-Degraded Image Generation

**Figure 2:**
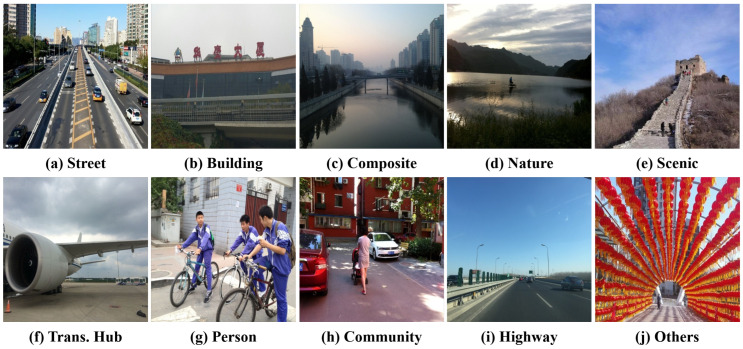
Ten scenes in the RMTD dataset.

**Figure 3:**
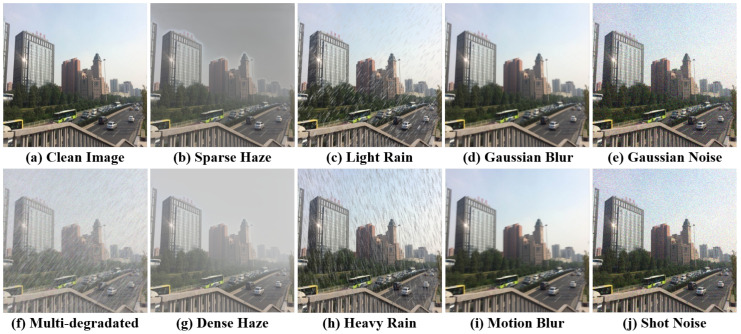
Degradation configurations in the RMTD dataset. The multi-degraded images include four types of degradation (haze, rain, blur, noise) simultaneously.

Synthetic multi-degraded images are generated from 3000 pristine clear outdoor images sourced from the Beijing-tour web [43]. These pristine images are categorized into 10 scenes (Figure 1 and Figure 2), ensuring a diverse representation of outdoor environments. Each image is degraded by 8 degradation types, grouped into four categories: haze, rain, blur, and noise (Figure 3).

**Figure 4:**
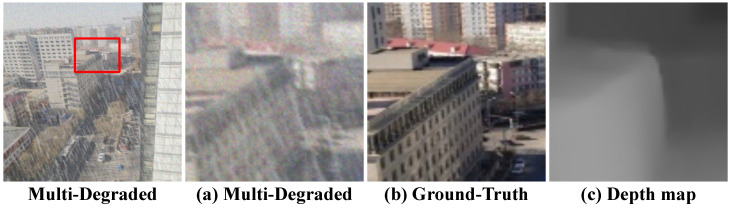
Illustration of a synthetic multi-degraded image with corresponding ground truth and depth map.

To simulate real-world conditions, each pristine image undergoes degradation by one of the four categories, with two settings per category, resulting in 16 unique degradation combinations per image. This approach captures both individual and compounded degradation effects, providing a comprehensive range of degradation scenarios for model evaluation. An example of a multi-degraded image, along with its ground truth and depth map, is shown in Figure 4.

#### 3.1.2. Haze Simulation

To realistically simulate hazy conditions, we adopt the physically grounded atmospheric scattering model [44]:

(1) I(x)=J(x)t(x)+A(1−t(x)),

where I(x) and J(x) represent the hazy image and the clean image, respectively, and *A* denotes atmospheric light. The transmission map is given by $t\left(x\right)=e^{-\beta d(x)}$, where β is the scattering coefficient of the atmosphere, and d(x) is the distance between the object and the camera. We calculate the distance using MegaDepth [45]. The parameters for light and dense haze are carefully chosen to span common real-world conditions: for light haze, A∈[0.5,0.7], β∈[0.6,1.0]; for dense haze, A∈[0.7,0.9], β∈[1.0,1.4]. These ranges simulate phenomena from mild mist to heavy fog, effectively replicating the visibility degradation caused by atmospheric particles.

#### 3.1.3. Rain Simulation

Rain is synthesized to mimic its complex appearance in real captures. We generate realistic rain streaks using the method from [3]:

(2) R=Trans(random(I),v,l),

where *I* is the input image, *R* is the rain mask, random(I) is a random noise map with the same size of input image, *v* is the minimal size of preserved rain, and *l* is the average length of rain streaks. Rainy images are computed as:

(3) O=I·(1−R)+(1−α)R,

where *O* is the rainy image, *I* is the pristine image, *R* is the rain mask, and α controls streak transparency.

Parameters are set to cover varied precipitation intensities: light rain uses v=2, l=10, α=0.8, simulating sparse, fine streaks; heavy rain uses v=5, l=15, α=0.6, producing denser, more opaque rain layers that significantly obscure visibility.

#### 3.1.4. Blur Simulation

We simulate two prevalent types of blur encountered in practical imaging.

Gaussian blur approximates the effect of improper focus or atmospheric turbulence, implemented via convolution with a Gaussian point-spread function (PSF) [46]:

(4) g(x,y)=(f×h)(x,y),

where *f* is the ideal scene, *h* is the point-spread function (PSF), and *g* is the observed image. The kernel severity σ is sampled from {0.2,0.4,0.6,0.8,1.0}, covering a range from slight to noticeable softness.

Motion blur mimics camera shake or object movement. We employ a heterogeneous kernel model [47]:

(5) $$Y\left(i,j\right)=\sum_{i^{\prime },j^{\prime }}K\left(i,j\right)\left(i^{\prime },j^{\prime }\right)X\left(i+i^{\prime },j+j^{\prime }\right),$$

where *X* denotes the sharp image and *K* denotes a heterogeneous motion blur kernel map with different blur kernels for each pixel in *X*. Parameters (radius ∈[6,9], σ∈[1.0,2.5]) are randomized to generate diverse blur directions and intensities, closely resembling real motion artifacts.

#### 3.1.5. Noise Simulation

We model two dominant noise types in digital imaging.

Gaussian noise arises from sensor heat and electronic interference, following the distribution:

(6) $$p\left(z\right)=\frac{1}{\sigma \sqrt{2\pi }}e^{-\frac{{\left(z-\mu \right)}^{2}}{2\sigma^{2}}},$$

where μ is the mean, and σ is the standard deviation. With μ=0 and σ∈[0.04,0.10], we simulate moderate to strong sensor noise prevalent in low-quality captures.

Shot noise (Poisson noise) stems from the photon-counting process in image sensors [48]:

(7) $$P\left(X=k\right)=\frac{e^{-\lambda }\lambda^{k}}{k!},$$

where λ is the average rate of events per interval, and *k* is the number of events. The severity parameter λ is chosen from {50,75,100,250,500}, emulating various illumination conditions from low-light (high noise) to well-lit scenarios (low noise).

#### 3.1.6. Collection of Real-World Degraded Images

The Real subset consists of 200 real-world images collected from various online sources (e.g., Google, Baidu) to reflect diverse degradation conditions, including varying intensities of haze, rain, blur, and noise. These images cover all 10 outdoor scene categories (as shown in Figure 1), ensuring a broad representation of real-world environmental scenarios.

Unlike the synthetic images, these real-world images do not have ground truth data, making them an essential resource for evaluating model performance under uncontrolled and complex real-world conditions. As there are no direct reference points for comparison, these images challenge models to generalize well in the absence of perfect information, thus reflecting real-world use cases where ground truth may not be available.

**Figure 5:**
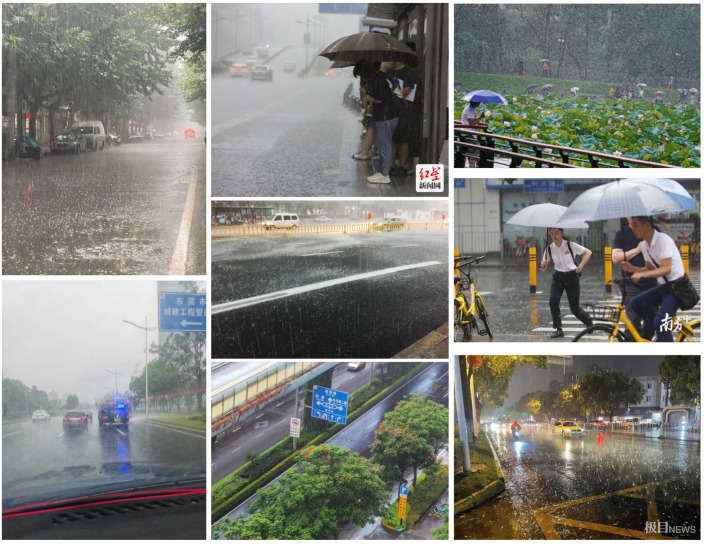
Visualization of real multi-degraded images sampled from RMTD-Real subset. The Chinese text in the image consists of watermarks and traffic signs.

For examples of real-world degraded images, refer to Figure 5, where representative samples from the Real subset are displayed.

#### 3.1.7. Object Detection Annotations

To enhance the dataset’s utility for downstream tasks, we incorporated object detection annotations into both the synthetic Test set and the real-world Real set. Annotations were created using the LabelImg tool [49] and saved in the PASCAL VOC format [50].

The dataset includes annotations for 10 object categories: car, truck, traffic light, person, motorbike, backpack, bus, handbag, bicycle, and umbrella. These categories are selected for their relevance to urban outdoor environments and applications like autonomous driving and urban surveillance.

**Figure 6:**
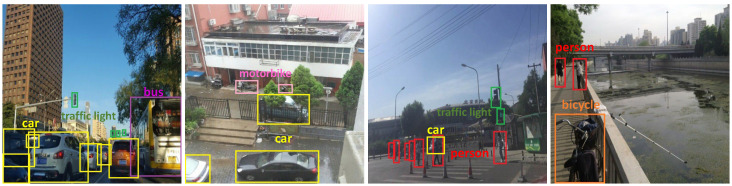
Visualization of object detection annotation boxes in outdoor scenes.

The annotations serve two key purposes: (1) evaluating how well image restoration models preserve object features after degradation and (2) assessing the performance of object detection models under diverse degradation conditions. By testing detection on restored images, we can gauge the impact of restoration techniques on downstream tasks. Examples of annotated images are shown in Figure 6.

### 3.2. Dataset Statistics and Analysis

#### 3.2.1. Scene Distribution

RMTD spans 10 outdoor scene categories: Street, Building, Composite, Nature, Scenic, Transportation Hub, Person, Community, Highway, and Others. As shown in Figure 1, the dataset is designed to reflect a broad range of real-world environments, with “Street”, “Building”, and “Composite” scenes dominating, reflecting the dataset’s urban focus. The inclusion of categories such as “Nature”, “Scenic”, and “Transportation Hub” ensures a diverse representation of outdoor environments. The “Others” category aggregates scenes that do not fit neatly into the other nine categories, ensuring further diversity and generalization across different environments.

#### 3.2.2. Object Annotation Statistics

**Figure 7:**
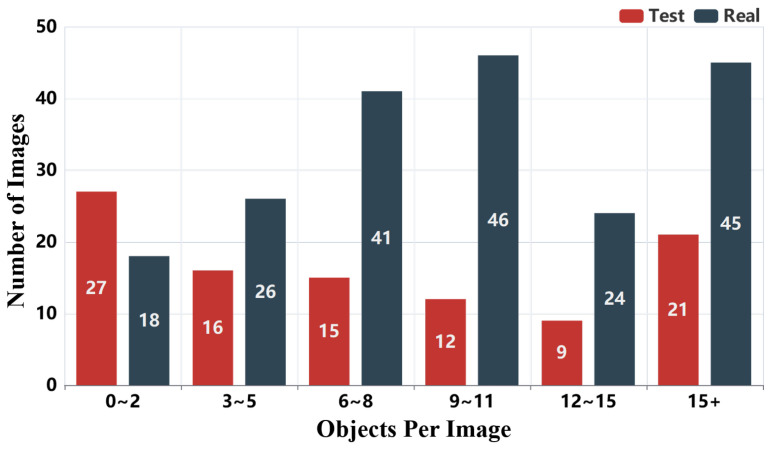
Distribution of object detection annotation boxes per image.

**Figure 8:**
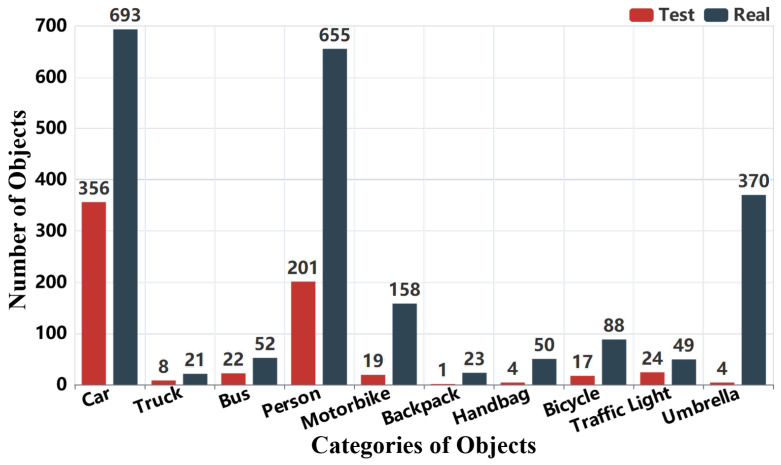
Category distribution of object detection annotation boxes.

The distribution of object annotations is shown in Figure 7 and Figure 8. In Figure 7, we observe that most images contain multiple annotated objects, with a significant number containing more than 15 object annotations. This highlights the dataset’s complexity and relevance for real-world applications. Figure 8 presents the distribution of 3000 object boxes, where “car” and “person” dominate, reflecting the urban and transportation focus of RMTD. This distribution of annotations is consistent with common objects encountered in outdoor environments like urban streets.

#### 3.2.3. Summary

RMTD addresses key limitations of prior datasets by combining synthetic and real-world images, supporting diverse degradation scenarios, and incorporating object detection annotations. These features make RMTD a comprehensive benchmark for evaluating robust image restoration techniques, particularly in urban environments, and for assessing the impact of restoration methods on downstream tasks like object detection.

## 4. Method

This section outlines the architecture of the proposed FFformer model, featuring novel components specifically designed for effective multi-degraded image restoration, including the GFSA, FSFN, SCM, FAM, and FEB.

### 4.1. Overall Pipeline

**Figure 9:**
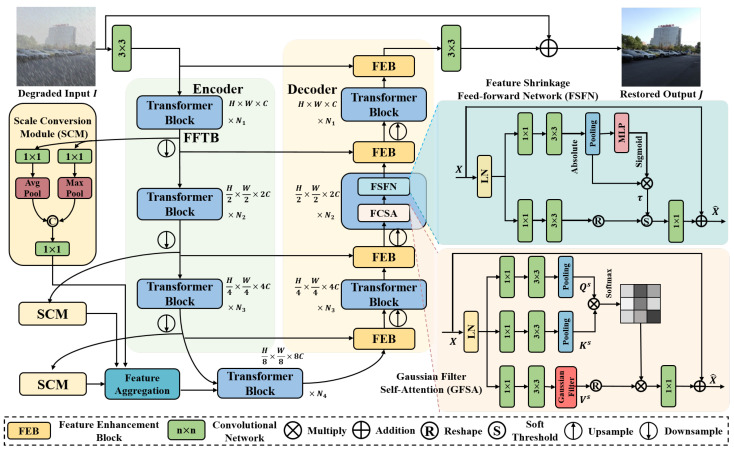
Overall architecture of the proposed FFformer for multi-degraded image restoration.

As illustrated in Figure 9, the architecture of FFformer is built upon a hierarchical transformer encoder–decoder framework aimed at tackling the challenges associated with multi-degraded image restoration. Given a degraded input $I\in R^{H\times W\times 3}$, FFformer initiates the process by extracting low-level features $X_{0}\in R^{H\times W\times C}$ using a 3×3 convolution, where *C* represents the initial channel size. These initial features serve as a foundational representation capturing essential information from the input.

The extracted features then proceed through a 4-level encoder–decoder structure, which serves as the backbone of the FFformer architecture. Each encoder and decoder module contains multiple Feature Filter Transformer Blocks (FFTBs), specialized components crucial for managing multiple degradations in images. Within each FFTB, conventional mechanisms such as Multi-head Self-Attention (MSA) and Feed-forward Networks (FN) are replaced with our proposed GFSA and FSFN. These enhancements not only improve the model’s ability to capture critical information and eliminate unnecessary degradations but also contribute to FFformer’s lightweight design.

In the encoding stage, each encoder progressively reduces the spatial dimensions by half while doubling the channel size, facilitating a hierarchical abstraction of features. Multi-scale features are then processed through an SCM to adapt the scales for optimal utilization. Aggregated features from various encoder layers are fed into the image decoder via an additional FAM, ensuring comprehensive feature utilization.

Conversely, the decoding stage reconstructs the spatial dimensions by twofold while halving the channel size, enabling the network to generate a restored image that faithfully captures essential details. Departing from standard skip connections [30,51], which typically concatenate encoder and decoder features, FFformer incorporates the FEB. This block plays a critical role in further extracting and preserving features essential for multi-degradation restoration.

To maintain a balance between computational efficiency and information preservation, pixel-unshuffle and pixel-shuffle operations [52] are strategically employed for down-sampling and up-sampling of features.

After the decoding stage, a residual image $R\in R^{H\times W\times 3}$ is generated through another 3×3 convolution, capturing the nuanced differences between the degraded input and the reconstructed features. Finally, the restored output is obtained as O=R+I. The entire network is trained by minimizing the $L_{1}$ norm loss:

(8) $$\begin{array}{c} L=∥O-I_{gt}{∥}_{1}, \end{array}$$

where $I_{gt}$ denotes the ground truth and ∥·∥ denotes the $L_{1}$ norm. This loss function ensures the convergence of the network towards accurate restoration.

### 4.2. Gaussian Filter Self-Attention

In the task of complex scene multi-degraded image enhancement, the presence of multiple compounded degradation factors not only leads to a significant decline in image visual quality but also results in the features extracted by image enhancement networks being heavily contaminated with complex and high-proportion degradation factors. Relying solely on the inherent learning capabilities of deep neural networks to distinguish composite degradation features is not only challenging but also time-consuming, thereby increasing the risk of network overfitting. To address this issue, FFformer introduces a GFSA mechanism, incorporating the widely used Gaussian filter for signal denoising and smoothing in images into the self-attention mechanism of the visual Transformer structure. This approach aims to partially remove composite degradation factors at the feature level, thereby reducing the learning difficulty for the network. The two-dimensional Gaussian function is presented in Equation (6).

A significant contributor to the computational overhead in Transformers is the key-query dot product interaction within the self-attention layer [53,54]. To address this challenge, GFSA involves max-pooling the key-query features to a fixed size of 8×8 and computing cross-covariance across channels rather than across spatial dimensions. This strategic approach results in an attention matrix of size $R^{C\times C}$, effectively alleviating the computational burden associated with traditional self-attention mechanisms.

The GFSA process begins with layer normalization, followed by 1×1 and 3×3 convolutions to prepare the input *X* for subsequent operations. To leverage both pixel-wise cross-channel and channel-wise spatial context, two max-pooling layers are employed. These layers not only retain local information but also ensure a fixed feature size for both the key and query. The GFSA process can be expressed as:

(9) $$\begin{array}{c} Q^{s}=Pool\left(Conv\left(X\right)\right),K^{s}=Pool\left(Conv\left(X\right)\right),V^{s}=GF\left(Conv\left(X\right)\right), \end{array}$$

(10) $$\begin{array}{c} \hat{X}=softmax\left(Q^{s}\cdot K^{s}/\lambda \right)V^{s}+X, \end{array}$$

where λ is an optional temperature factor defined by $\lambda =\sqrt{d}$.

The integration of Gaussian filtering within the self-attention mechanism is motivated by its frequency-domain properties. The Gaussian filter in the spatial domain is defined as:

(11) $$G\left(x,y\right)=\frac{1}{2\pi \sigma^{2}}\exp^{-\frac{x^{2}+y^{2}}{2\sigma^{2}}},$$

where (x,y) are spatial coordinates and σ is the standard deviation determining the filter’s width. Through the Fourier transform, its representation in the frequency domain maintains a Gaussian shape:

(12) $$G\left(u,v\right)=\exp^{-2\pi \sigma^{2}\left(u\right)},$$

where (u,v) are frequency-domain coordinates. The magnitude–frequency characteristic of the Gaussian filter is, therefore, a Gaussian function, which exhibits low-pass filtering properties.

In image processing, low-frequency components generally correspond to the primary structural information of an image, while high-frequency components often encompass noise and fine-texture details. By incorporating a Gaussian filter into the self-attention mechanism, we selectively attenuate high-frequency components in the feature maps. This operation directly affects the attention distribution by smoothing the calculated attention scores, making the model less sensitive to high-frequency noise and local perturbations. Consequently, it guides the model to focus on broader, more semantically consistent regions, thereby stabilizing the training process and enhancing feature quality by emphasizing robust, low-frequency information. This formulation captures the essence of the GFSA mechanism, demonstrating its ability to enhance the attention computation process while preserving the input features. The introduction of GFSA not only reduces computational overhead but also facilitates the efficient capture of essential information.

### 4.3. Feature Shrinkage Feed-Forward Network

While previous studies [30,55] have integrated depth-wise convolutions into feed-forward networks to enhance locality, they often overlook the inherent redundancy and noise in features critical for multi-degraded image restoration. To address this gap, we introduce the FSFN, a novel mechanism that employs a soft-threshold function to shrink feature values. This strategic approach aims to significantly reduce redundancy and eliminate unwanted degradations, thereby ensuring the robustness of the restoration process.

As depicted in Figure 9, the FSFN process begins with the input *X*, which undergoes normalization and convolution operations to prepare it for subsequent feature shrinkage. The average score *M* of size $R^{1\times 1\times C}$ is calculated as M=GPool(|Conv(X)|), where GPool represents global average pooling. The threshold τ is then defined as τ=M·sigmoid(MLP(M)). The overall FSFN process can be expressed as:

(13) $$\begin{array}{c} \hat{X}=SThold\left(Conv\left(LN\left(X\right)\right)\right)+X, \end{array}$$

where SThold(x) is a soft-thresholding function defined as:

(14) $$\begin{array}{c} SThold\left(x\right)=\left\{\begin{array}{cc} x-\tau & x>\tau \\ 0 & -\tau \leq x\leq \tau \\ x+\tau & x<-\tau \end{array}\right. \end{array}$$

The theoretical motivation for this operation is twofold:

- Sparsity Promotion: The soft-thresholding function zeros out all feature elements whose absolute values are below the threshold τ. This actively promotes sparsity in the feature representation, effectively filtering out a large number of weak or non-significant activations that are likely to be noise or less informative components.
- Noise Reduction: In signal processing theory, soft-thresholding is known to be the proximal operator for the L1-norm and is optimal for denoising signals corrupted by additive white Gaussian noise. By applying this principle to feature maps, the FSFN module acts as an adaptive feature denoiser. It shrinks the magnitudes of all features, aggressively pushing insignificant ones (presumed noise) to zero while preserving the significant ones (presumed signal).

Unlike the simple gating mechanisms in the Depthwise Feed-forward Network (DFN) [55], soft-thresholding provides a principled, data-driven approach to noise removal and feature selection. While DFN [55] primarily perform smoothing or weighting, soft-thresholding implements an explicit “shrink or kill” strategy, which is theoretically grounded in sparse coding and leads to a more robust and compact feature representation, particularly effective in the presence of complex, real-world degradations. The incorporation of FSFN within the FFformer framework significantly reduces redundancy, facilitating the effective elimination of degradations.

### 4.4. Scale Conversion Module and Feature Aggregation Module

To effectively harness the multi-scale features generated during the encoding process of FFformer, we introduce two key components: the SCM and the FAM. These modules are essential for enhancing feature representations and facilitating coherent integration across different scales.

The Scale Conversion Module is designed to resize feature representations while preserving critical information. It initiates the process by applying both max pooling and average pooling to the input feature $F_{e}^{i}$, reducing its spatial dimensions to H/8×W/8. This reduction captures important characteristics at a lower resolution, allowing for more efficient processing. The operations are defined as follows:

(15) $$\begin{array}{c} F_{m}^{i}=MaxPool\left(Conv\left(F_{e}^{i}\right)\right), \end{array}$$

(16) $$\begin{array}{c} F_{a}^{i}=AvgPool\left(Conv\left(F_{e}^{i}\right)\right). \end{array}$$

The outputs from both pooling operations are then concatenated and processed through another 1×1 convolution to expand the channel size to 8C:

(17) $$\begin{array}{c} F_{k}^{i}=Conv_{1}\left(Concat\left(F_{m}^{i},F_{a}^{i}\right)\right). \end{array}$$

This concatenation enriches the feature set by integrating both local and averaged information, ultimately enhancing feature representation.

Following the SCM, the FAM is employed to consolidate the processed features. The FAM consists of two 1×1 convolutional layers and incorporates a channel-wise self-attention mechanism to capture long-range dependencies within the feature maps. This integration is performed as follows:

(18) $$\begin{array}{c} F_{u}=Conv\left(Concat\left(F_{k}^{i}\right)\right),i=1,2,3, \end{array}$$

(19) $$\begin{array}{c} \hat{F_{u}}=Conv\left(CSA\left(F_{u},F_{u},F_{u}\right)\right)+F_{u}, \end{array}$$

where $CSA\left(Q,K,V\right)=softmax\left(Q\cdot K^{⊤}/\lambda \right)V$ denotes the channel-wise self-attention operation, with λ as a learnable scaling factor. This attention mechanism selectively emphasizes significant features while downplaying irrelevant ones, enhancing the module’s overall efficacy. Additionally, the integration of residual connections promotes stability and convergence during the learning process, further improving the performance of the module.

### 4.5. Feature Enhancement Block

The effectiveness of skip connections in U-Net-like architectures is often hampered by the semantic gap and spatial misalignment between encoder and decoder features. While prevalent feature enhancement modules, such as channel or spatial attention mechanisms, primarily focus on refining features within a single pathway, they are less effective in dynamically calibrating and fusing features from two distinct pathways (i.e., the decoder and the residual connection). To address this limitation, we propose the FEB. The key innovation of FEB lies in its dual-attention guided feature calibration and fusion mechanism, which explicitly and simultaneously models both cross-feature and intra-feature dependencies to achieve more effective skip connections.

**Figure 10:**
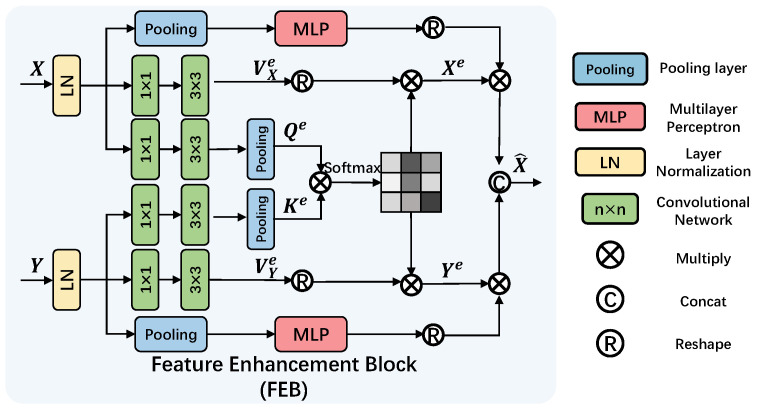
Implementation of the proposed FEB.

Unlike traditional skip connections [30,51] that simply concatenate encoder and decoder outputs, the FEB introduces a more sophisticated approach to extracting and preserving critical features within a residual framework. As depicted in Figure 10, the FEB incorporates both cross-feature channel attention and intra-feature channel attention mechanisms, strategically designed to enhance the model’s capability to utilize features effectively.

Given a decoder feature *X* and a residual feature *Y*, the FEB initiates by computing the query, key, and value matrices for cross-feature attention. To distinguish these from the self-attention matrices in GFSA, we denote them with the superscript e (for “enhancement”): $Q^{e}=Pool\left(Conv\left(X\right)\right)$, $K^{e}=Pool\left(Conv\left(Y\right)\right)$, $V_{X}^{e}=Conv\left(X\right)$, and $V_{Y}^{e}=Conv\left(X\right)$. These computations facilitate the subsequent cross-feature channel attention mechanism. Similar to GFSA, the intermediate outputs are calculated as $X^{e}=\mathrm{softmax}\left(Q^{e}\cdot K^{e}/\lambda \right)V_{X}^{e}$ and $Y^{e}=\mathrm{softmax}\left(K^{e}\cdot Q^{e}/\lambda \right)V_{Y}^{e}$.

The FEB further incorporates intra-feature channel attention by applying global average pooling to each feature map, generating an attention matrix. The overall formulation of the FEB process is given by:

(20) $$\begin{array}{c} \hat{X}=Concat\left(X^{e}\cdot MLP\left(Gpool\left(LN\left(X\right)\right)\right).Y^{e}\cdot MLP\left(Gpool\left(LN\left(Y\right)\right)\right)\right). \end{array}$$

This formulation captures the essence of the Feature Enhancement Block, highlighting its ability to synergistically combine cross-feature and intra-feature channel attention for improved extraction and preservation of vital features. By integrating the FEB within the residual structure, the model transcends basic concatenation, offering a refined and effective mechanism for enhancing crucial feature representation.

## 5. Experiments and Analysis

In this section, we provide a comprehensive comparison between our proposed FFformer and other state-of-the-art methods designed for task-specific and multi-degradation removal.

### 5.1. Datasets

Our experiments utilize the newly introduced RMTD dataset alongside subsets of the BID2a dataset, specifically the 5th (BID2a-5) and 6th (BID2a-6) subsets, as outlined by Han et al. [8]. The BID2a-5 dataset consists of synthetic images affected by rain and haze, while the BID2a-6 dataset presents the additional challenge of simultaneous disturbances from rain, haze, and snow. These datasets serve as valuable benchmarks for assessing the robustness and versatility of image restoration methods under various weather conditions.

### 5.2. Implementation Details

Our framework is implemented using PyTorch 1.10. For RMTD dataset, the models are trained on the Train subset, validated on 1600 images from the Others subset, and evaluated on the dedicated RMTD Test subset. This split ensures that the model is tuned and assessed on distinct data sources. We use the Adam optimizer with parameters ($\beta_{1}=0.9$, $\beta_{2}=0.999$) and a weight decay of $1\times 10^{-4}$. The models are trained for 200 epochs with a batch size of 8. The training loss is the L1 loss between the predicted and ground-truth images. The initial learning rate is set to $1\times 10^{-4}$ and is gradually reduced to $1\times 10^{-6}$ using a cosine annealing scheduler [56] with a period of 200 epochs (T-max = 200) and no restarts. Input images are randomly cropped to a fixed patch size of 256×256 during training, while center cropping is applied for validation and testing. The architectural hyperparameters are set as follows: the number of FFformer blocks $N_{1},N_{2},N_{3},N_{4}$ is 2,4,4,6, the number of attention heads in the GFSA modules is 1,2,4,8, and the base channel dimensions are 32,64,128,256.

### 5.3. Evaluation Metrics

To assess the performance of multi-degraded image restoration on labeled datasets RMTD-Syn, BID2a-5 [8], and BID2a-6 [8], we employ two widely used full-reference metrics: Peak Signal-to-Noise Ratio (PSNR in dB) [57] and Structural Similarity Index (SSIM) [58]. These metrics provide a quantitative assessment by comparing the restoration results with the corresponding ground truth images.

Additionally, we utilize two non-reference image quality evaluation indicators: BRISQUE [59], which measures potential losses of naturalness in images, and NIQE [60], which is based on a collection of statistical features constructed from a space-domain natural scene statistic (NSS) model.

### 5.4. Comparisons with State-of-the-Art Methods

We conduct a comprehensive comparison between FFformer and state-of-the-art restoration methods. The evaluated methods include four task-specific approaches (DerainNet [3], Principled-Synthetic-to-Real-Dehazing (PSD) [61], Complementary Cascaded Network (CCN) [62], Deblur-NeRF [63]) and seven generalized restoration methods (MPRNet [28], Uformer [30], Restormer [35], BIDeN [8], Weather-General and Weather-Specific (WGWS) [32], Patil et al. [31], Adaptive Sparse Transformer (AST) [33]). More details about the comparison methods are provided in Table 2.

**Figure 11:**
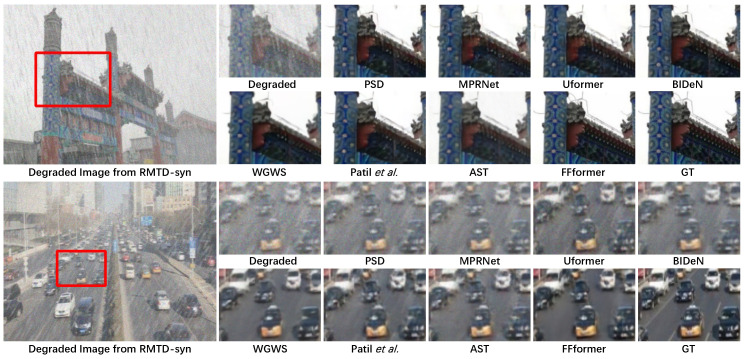
Qualitative restoration results on the RMTD-Syn dataset with PSD [61], MPRNet [28], Uformer [30], BIDeN [8], WGWS [32], Patil et al. [31], and AST [33].

**Figure 12:**
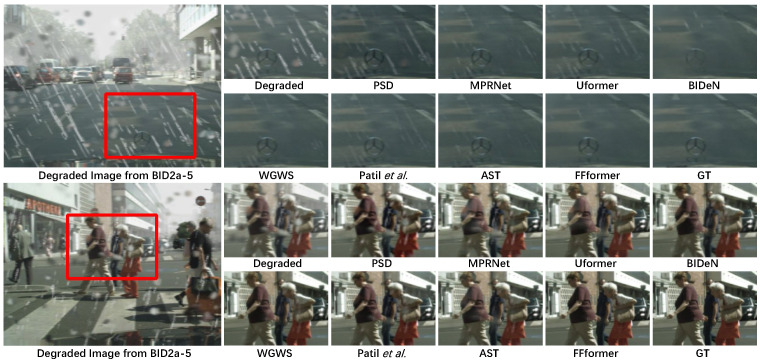
Qualitative restoration results on the BID2a-5 [8] with PSD [61], MPRNet [28], Uformer [30], BIDeN [8], WGWS [32], Patil et al. [31], and AST [33].

**Figure 13:**
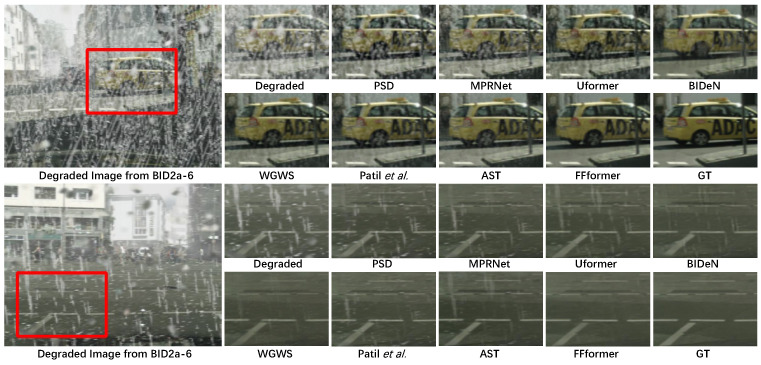
Qualitative restoration results on BID2a-6 [8] with PSD [61], MPRNet [28], Uformer [30], BIDeN [8], WGWS [32], Patil et al. [31], and AST [33].

Synthetic. Qualitative assessments on synthetic datasets are visually demonstrated in Figure 11, Figure 12 and Figure 13. FFformer exhibits impressive capabilities in removing multiple degradations, producing high-quality images that closely resemble the ground truth. The quantitative results, as shown in Table 3 and Table 4, highlight FFformer’s consistent superiority over other methods, with PSNR and SSIM scores of 28.67 vs. 28.24 and 0.880 vs. 0.873, respectively, on the RMTD-Syn dataset. The satisfactory results obtained by FFformer on BID2a-5 and BID2a-6 further affirm its efficacy in restoring multi-degraded images across diverse synthetic datasets. This robust and versatile performance underscores FFformer’s effectiveness in tackling the challenges posed by multi-degradations, solidifying its position as a reliable solution for various multi-degraded image restoration scenarios.

Real. To thoroughly evaluate the model’s performance and generalization capability in authentic, uncontrolled outdoor scenarios, we conducted extensive experiments on the RMTD-Real. Furthermore, to directly address the model’s ability to generalize across unseen degradation domains, we designed a cross-dataset validation experiment.

The quantitative results are summarized in Table 5. Crucially, the new Table 5 provides a cross-dataset analysis, where models were trained on different source datasets—BID2a, BID2b, and our RMTD-Syn—and evaluated on the target RMTD-Real set. The results confirm two key findings: First, training on our RMTD-Syn dataset yields the best performance on real-world images, even when compared to models trained on other real-world degradation datasets (BID2b). This demonstrates that the comprehensive multi-degradation simulation in RMTD effectively bridges the sim-to-real gap. Second, our FFformer consistently achieves the best no-reference quality metrics, attesting to its robustness and adaptability.

**Figure 14:**
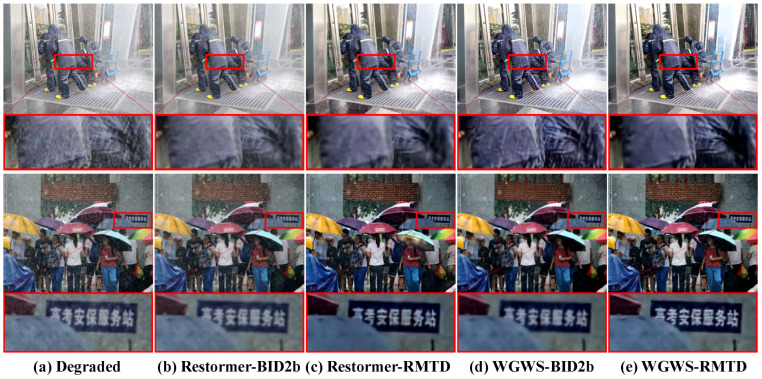
Visual comparison of models trained on different datasets and evaluated on RMTD-Real. The comparison between (**b**,**d**) models trained on BID2b and (**c**,**e**) models trained on RMTD-Syn demonstrates that training on our diverse synthetic dataset yields superior restoration of details and more effective degradation removal in complex real-world conditions. The image contains a Chinese sign, which translates to “Gaokao Security Service Station”.

Qualitatively, Figure 14 provides a compelling visual comparison. It shows that models trained on the weather-specific BID2b dataset struggles to fully restore a real-world image from RMTD-Real, which likely contains a complex mixture of degradations beyond just weather. In contrast, models trained on our diverse RMTD-Syn dataset successfully removes artifacts and recovers finer details.

**Figure 15:**
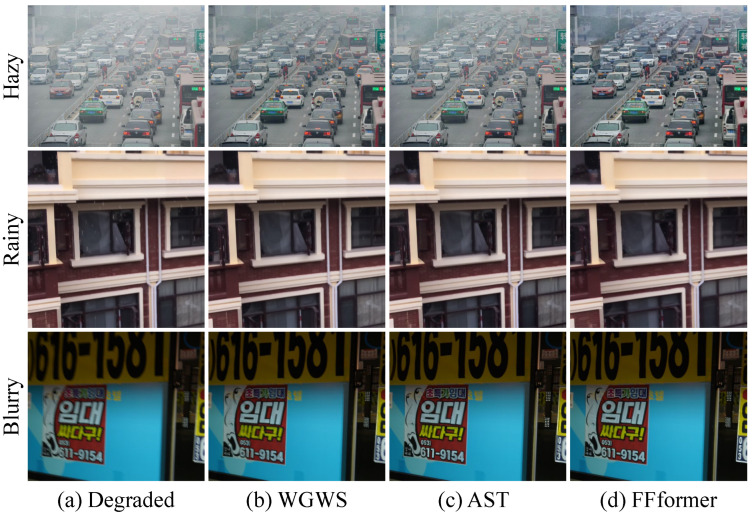
Cross-dataset generalization to real-world images with single degradations. All compared models were trained solely on the proposed RMTD-Syn dataset (multi-degradation) but are evaluated here on real images from external sources, each exhibiting a single dominant degradation (Hazy, Rainy, Blurry). The successful restoration across these different degradation domains demonstrates the strong generalization capability and robust feature learning fostered by our training dataset. Furthermore, our FFformer achieves the most visually pleasing results with the cleanest backgrounds and best-preserved details. The image contains a Korean advertisement poster implying low-cost rentals.

Finally, Figure 15 provides a critical analysis of the model’s cross-dataset generalization capability. It showcases the restoration results of different models (all trained on our multi-degradation RMTD-Syn) when applied to real-world images from external sources (RTTS [1], SPA [4], BLUR-J [6]), each characterized by a single, dominant degradation type. The compelling performance across these diverse degradation domains demonstrates that the feature representations learned from our comprehensive RMTD-Syn dataset are highly robust and generalizable, effectively transferring to restoration tasks beyond the specific multi-degradation mixtures seen during training. Among them, our FFformer consistently produces the most visually pleasing results with the cleanest backgrounds and best-preserved details, solidifying its status as a robust and versatile solution for real-world image restoration.

Object Detection. The assessment of object detection performance, conducted using YOLOv8 [65] on restoration results, highlights FFformer’s consistent superiority in accurately detecting objects within multi-degraded images. As shown in Table 6, FFformer outperforms alternative methods, demonstrating its exceptional ability to restore images while preserving crucial details necessary for reliable object detection. This comprehensive evaluation underscores FFformer’s efficacy and robustness in restoring images affected by complex multi-degradations, positioning it as a state-of-the-art solution for multi-degraded image restoration tasks.

### 5.5. Ablation Studies

**Figure 16:**
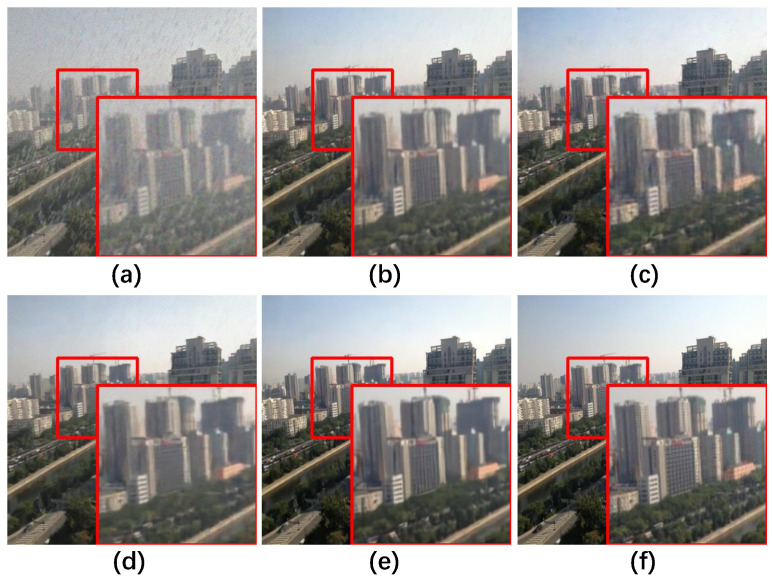
Qualitative ablation study results of the Feature Filter Transformer Block on RMTD-Syn dataset. (**a**) Degraded, (**b**) MSA + FN, (**c**) GFSA + FN, (**d**) MSA + FSFN, (**e**) GFSA + FSFN, (**f**) Ground Truth.

The ablation study of the transformer architecture is summarized in Table 7 and Figure 16. The GFSA, which focuses on selecting local maximum features, outperforms the MSA, achieving a notable 0.26 dB gain in PSNR and a 0.007 gain in SSIM. Additionally, the feature value shrinkage introduced by the FSFN enhances its ability to filter redundant degradations, resulting in a 0.27 dB PSNR gain over the conventional FN [54] and a 0.08 dB PSNR gain over the DFN [55]. Overall, compared to the baseline, the architecture achieves a significant improvement with a 0.82 dB gain in PSNR and a 0.018 gain in SSIM.

**Figure 17:**
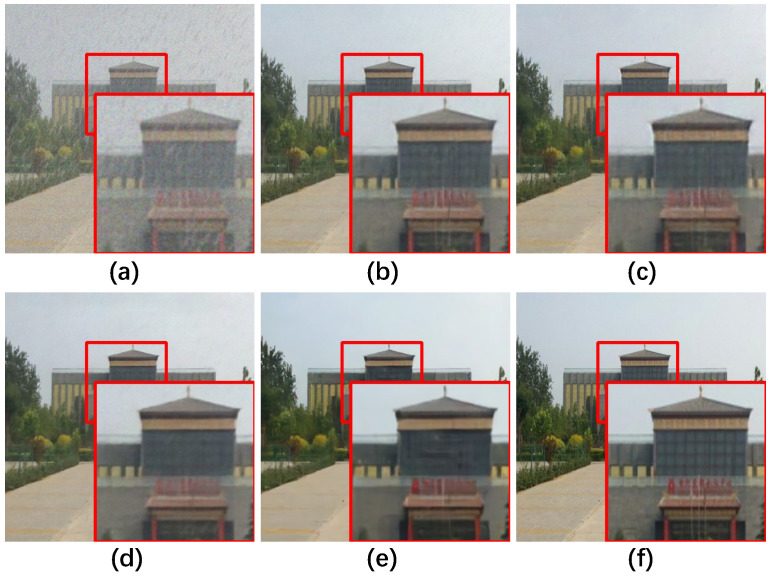
Qualitative ablation study results of the Feature Enhancement Block on RMTD-Syn dataset. (**a**) Degraded, (**b**) w/o FEB, (**c**) w/o intra-feature attention, (**d**) w/o cross-feature attention, (**e**) intra-feature + cross-feature attention, (**f**) Ground Truth.

The ablation study results presented in Table 8 and Figure 17 further illuminate the significant contribution of the FEB to the overall network improvement. FEB plays a crucial role, leading to a remarkable enhancement of 0.76 dB in PSNR and a substantial gain of 0.022 in SSIM. This underscores the effectiveness of FEB in refining and enriching feature representations, significantly contributing to FFformer’s restoration performance.

### 5.6. Study of Hyper-Parameters and Model Complexity

In this study, we investigate the impact of hyper-parameters and model complexity on the performance of FFformer. Four hyper-parameter configurations are tested, varying layer numbers, attention heads, and channel numbers. Specifically, we consider two settings for layer numbers: {4, 4, 4, 4} and {2, 4, 4, 6}, along with two settings for attention heads: {2, 2, 4, 4} and {1, 2, 4, 8}. The corresponding channel numbers are {64, 64, 128, 128} and {32, 64, 128, 256}, respectively. The comparison results are summarized in Table 9.

**Figure 18:**
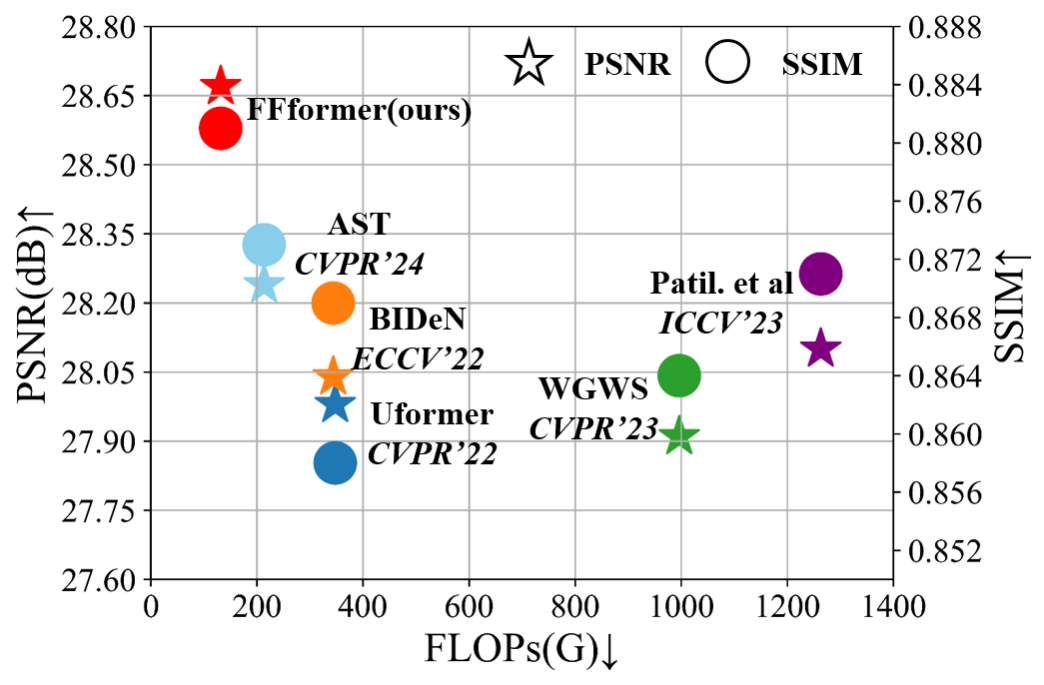
PSNR↑ and SSIM↑ vs. FLOPs↓ on the RMTD. FFformer outperforms state-of-the-art methods (AST [33] in cyan, BIDeN [8] in orange, Uformer [30] in blue, WGWS [32] in green, and Patil et al. [31] in purple) in both metrics while maintaining lower computational complexity.

Furthermore, the model complexity analysis in Table 10 reinforces FFformer’s position as a lightweight model. It not only exhibits the lowest computational complexity but also achieves the fastest average inference time on 512×512 pixel images. This efficiency is attributed to the synergistic effects of feature size reduction and feature value shrinkage introduced by GFSA and FSFN. As shown in Figure 18, the FFformer is an efficient and lightweight image enhancement model for complex scene images. Consequently, FFformer excels in restoration performance and proves to be a practical and efficient solution for multi-degraded image restoration tasks, making it suitable for applications in systems such as autonomous driving and safety monitoring.

## 6. Conclusions

In conclusion, this paper introduces the Feature Filter Transformer (FFformer) as a specialized solution for multi-degraded image restoration. By leveraging the synergistic capabilities of the Gaussian Filter Self-Attention (GFSA) and Feature Shrinkage Feed-forward Network (FSFN), FFformer effectively compresses feature sizes and shrinks feature values simultaneously. Additionally, FFformer employs the innovative Scale Conversion Module (SCM) and Feature Aggregation Module (FAM) to adeptly handle multi-scale features within the image encoder. The integration of the Feature Enhancement Block (FEB) further refines the extraction of valuable multi-degradation features in the decoder.

Furthermore, we present the inaugural Robust Multi-Type Degradation (RMTD) dataset, a significant milestone in image restoration methodologies, as it encompasses multiple degradations simultaneously. The creation of RMTD represents a crucial advancement, providing a valuable resource for ongoing research and future developments in the field. Comparative experiments conducted on the RMTD dataset and other sources compellingly demonstrate FFformer’s superior performance in multi-degraded image restoration. Ultimately, FFformer emerges as an innovative approach, promising robust solutions for applications reliant on accurate visual information under challenging weather conditions.

Looking ahead, our future work will focus on two key directions. First, we plan to extend our efficient restoration framework to enable comprehensive benchmarking against state-of-the-art large-scale generative and foundation models, which will require access to elevated computational resources. Second, we aim to continually expand the diversity and realism of the RMTD dataset by incorporating a wider spectrum of challenging real-world degradations.

## Figures and Tables

**Table 1:** Comparison of dataset statistics and characteristics between RMTD and existing benchmarks.

Dataset	Synthetic or Real	Number	Degradation Types	Real Test	Annotation
SIDD [7]	Real	30,000	Noise	Yes	No
RealBlur [6]	Real	4556	Blur	Yes	No
Rain1400 [3]	Synthetic	14,000	Rain	No	No
SPA [4]	Real	29,500	Rain	Yes	No
RESIDE [1]	Synthetic + Real	86,645 + 4322	Haze	Yes	RTTS
BID2a [8]	Synthetic	3475	4 types	No	No
BID2b [8]	Synthetic + Real	3661 + 1763	3 for train, 1 for test	Yes	No
RMTD	Synthetic + Real	48,000 + 200	8 types	Yes	Test, Real

**Table 2:** Details of the comparison methods.

Category	Methods	Source
Task-specific Methods	DerainNet [64]	TIP’ 2017
	PSD [61]	CVPR’ 2021
	CCN [62]	CVPR’ 2021
	Deblur-NeRF [63]	CVPR’ 2022
Multiple Degradations Removal Methods	MPRNet [28]	CVPR’ 2021
	Uformer [30]	CVPR’ 2022
	Restormer [35]	CVPR’ 2022
	BIDeN [8]	ECCV’ 2022
	WGWS [32]	CVPR’ 2023
	Patil et al. [31]	ICCV’ 2023
	AST [33]	CVPR’ 2024

**Table 3:** PSNR ↑/SSIM↑ on three multiple degradation removal datasets. ↑ denotes that a higher value indicates better performance.

Datasets	BID2a-5 [8]	BID2a-6 [8]	RMTD-Syn
**Degraded**	**14.05/0.616**	**12.38/0.461**	**15.09/0.410**
DerainNet [64]	18.68/0.805	17.53/0.721	21.43/0.772
PSD [61]	22.17/0.855	20.57/0.809	26.97/0.839
CCN [62]	20.86/0.831	19.74/0.782	25.41/0.830
Deblur-NeRF [63]	21.10/0.840	20.12/0.797	26.87/0.843
MPRNet [28]	21.18/0.846	20.76/0.812	27.31/0.860
Uformer [30]	25.20/0.880	25.14/0.850	27.98/0.858
Restormer [35]	25.24/0.884	25.37/0.859	28.02/0.868
BIDeN [8]	27.11/0.898	26.44/0.870	28.04/0.869
WGWS [32]	26.87/0.899	25.89/0.856	27.91/0.864
Patil et al. [31]	26.55/0.884	26.20/0.861	28.10/0.871
AST [33]	27.15/0.901	26.32/0.865	28.24/0.873
FFformer (ours)	**27.41**/**0.905**	**26.51**/**0.871**	**28.67**/**0.880**

**Table 4:** No-reference BRISQUE↓/NIQE↓ on three multiple degradation removal datasets. ↓ denotes that a lower value indicates better performance.

Datasets	BID2a-5 [8]	BID2a-6 [8]	RMTD-Syn
**Degraded**	**34.420/5.793**	**33.574/6.150**	**32.436/9.917**
DerainNet [64]	33.877/5.742	32.015/6.062	30.617/4.967
PSD [61]	34.876/5.665	33.116/6.156	29.317/4.101
CCN [62]	33.624/5.736	34.394/6.134	30.261/4.575
Deblur-NeRF [63]	31.733/5.695	31.531/5.864	29.411/4.038
MPRNet [28]	31.348/5.567	32.377/5.969	28.518/3.950
Uformer [30]	30.627/5.324	31.001/5.599	29.690/4.183
Restormer [35]	29.137/5.346	30.482/5.504	30.234/3.870
BIDeN [8]	27.967/5.242	28.395/5.386	28.043/3.902
WGWS [32]	28.495/5.201	28.897/5.431	27.917/3.824
Patil et al. [31]	28.365/5.197	28.172/5.354	27.710/3.833
AST [33]	28.144/5.166	27.814/5.301	27.967/3.764
FFformer (ours)	**27.134**/**5.084**	**26.313**/**5.146**	**26.451**/**3.685**

**Table 5:** Cross-dataset generalization evaluation on the RMTD-Real test set. Models are trained on different source datasets (BID2a, BID2b, RMTD-Syn) and evaluated on the target RMTD-Real set using no-reference image quality metrics (BRISQUE↓/PIQE↓). Results demonstrate the superior effectiveness of the proposed RMTD-Syn dataset for generalizing to real-world multi-degradation scenarios and the robust performance of our FFformer.

Training Set	BID2a [8]	BID2b [8]	RMTD-Syn
**Degraded**	**28.443/3.850**	**28.443/3.850**	**28.443/3.850**
DerainNet [64]	29.431/3.871	29.991/3.955	29.624/3.844
PSD [61]	27.970/3.860	27.317/3.851	27.246/3.717
CCN [62]	28.412/3.812	27.961/3.974	28.791/3.901
Deblur-NeRF [63]	28.011/3.784	29.411/3.978	27.664/3.695
MPRNet [28]	27.318/3.759	27.118/3.801	26.417/3.672
Uformer [30]	28.682/3.647	29.013/3.661	27.011/3.590
Restormer [35]	27.302/3.756	27.034/3.720	26.181/3.604
BIDeN [8]	26.704/3.688	26.430/3.667	25.448/3.506
WGWS [32]	25.813/3.670	25.517/3.657	24.682/3.547
Patil et al. [31]	25.710/3.667	25.613/3.671	24.827/3.598
AST [33]	26.124/3.733	25.867/3.715	24.961/3.568
FFformer (ours)	**25.012**/**3.562**	**25.334**/**3.541**	**23.437**/**3.427**

**Table 6:** Object detection results in mAP↑ using YOLOv8 [65].

Datasets	RMTD-Syn	RMTD-Real
Degraded	0.1580	0.5259
Uformer [30]	0.3710	0.5789
BIDeN [8]	0.3804	0.5893
Patil et al. [31]	0.3821	0.5876
AST [33]	0.3841	0.5955
FFformer	**0.3965**	**0.6012**
Ground Truth	0.4153	-

**Table 7:** Quantitative ablation study results of the Feature Filter Transformer Block on RMTD-Syn dataset.

Network	Component	PSNR ↑	SSIM ↑
baseline	MSA + FN [54]	27.85	0.862
Multi-head Attention	GFSA + FN [54]	28.11	0.869
Feed-forward Network	MSA + DFN [55]	28.04	0.867
	MSA + FSFN	28.12	0.871
Overall	GFSA + FSFN	**28.67**	**0.880**

**Table 8:** Quantitative ablation study results of the Feature Enhancement Block on RMTD-Syn dataset.

Setting	Cross-Feature Attention	Intra-Feature Attention	PSNR↑	SSIM ↑
(a)			27.91	0.858
(b)	✓		28.29	0.869
(c)		✓	28.17	0.865
(d)	✓	✓	**28.67**	**0.880**

**Table 9:** Comparison of Hyper-parameters on RMTD-Syn dataset.

Settings	Layer Nums	Attention Heads	PSNR↑/SSIM↑
(a)	4, 4, 4, 4	2, 2, 4, 4	26.97/0.862
(b)	4, 4, 4, 4	1, 2, 4, 8	28.10/0.868
(c)	2, 4, 4, 6	2, 2, 4, 4	28.17/0.867
(d)	2, 4, 4, 6	1, 2, 4, 8	**28.67**/**0.880**

**Table 10:** Comparison of Model Complexity.

Model	FLOPs	Parameters	Inference Time
Uformer [30]	347.6 G	50.9 M	0.1737 s
BIDeN [8]	344.0 G	38.6 M	1.2140 s
WGWS [32]	996.2 G	12.6 M	0.1919 s
Patil et al. [31]	1262.9 G	11.1 M	0.1098 s
AST [33]	213.6 G	13.4 M	0.1594 s
FFformer	131.7 G	17.2 M	0.0847 s

## Data Availability

Dataset available on request from the author.
